# Supplementary material for: Effectiveness of a quality improvement intervention to increase adherence to key practices during female sterilization services in Chhattisgarh and Odisha states of India
Source: PLoS One. 2020 Dec 23;15(12):e0244088. doi: 10.1371/journal.pone.0244088 (PMC7757870; doi:10.1371/journal.pone.0244088)
Supplement: S1 Checklist — (DOCX) [file pone.0244088.s001.docx]

**Assessment Checklist for Quality of Family Planning Study**

*Confidential (Information collected for research purpose only)*

|  | | | | | | |
| --- | --- | --- | --- | --- | --- | --- |
| **Name of the Observer :** | | | | | | |
| Observation date | | | | **DD** | **MM** | **YYYY** |
| Name of health facility …………………………… | | Block ……………………………………… | | | | |
| District: ……………………………. | | State……………………………. | | | | |
|  | |  | | | | |
| **Was checklist used in the facility** | | |  | | | |
| Pause Point 1 | | | 🞏 Yes 🞏 No | | | |
| Pause Point 2 | | | 🞏 Yes 🞏 No | | | |
| Pause Point 3 | | | 🞏 Yes 🞏 No | | | |
| Pause Point 4 | | | 🞏 Yes 🞏 No | | | |
| **Pause Point 1 (On admission)** | | | | | | |
| **Provider IDs -** | | **Client ID -** | | | | |
| **Sr. No.** | **Practice** | | | **Response** | | |
| **PP1.1** | **Client’s eligibility for undergoing sterilization procedure is checked and followed by service provider as per guidelines.** | | |  | | |
| 1 | Does the provider ask the client if she is fasting for at-least six hours today? | | | 🞏 Yes 🞏 No | | |
| 2 | Client’s vitals and investigations are measured and documented?  Vitals  🞏 Pulse 🞏 Blood pressure 🞏 Weight  Investigations  🞏 Hb 🞏 Urine (Albumin & Sugar) | | | 🞏 Yes 🞏 No | | |
| 3 | Client’s assessment has been done?  🞏 Medical status 🞏 Mental status 🞏 Abdominal examination  🞏 Pelvic examination | | | 🞏 Yes 🞏 No | | |
| **PP 1.2** | **Provider treats the client with respect**  ***Observe and note down whether the service provider:*** | | | | | |
| 1 | Interacts directly with client (not through any other person) and treats her respectfully. | | | 🞏 Yes 🞏 No | | |
| 2 | Briefly explains the procedure to the client and encourages her to ask questions/express concerns. | | | 🞏 Yes 🞏 No | | |
| **PP 1.3** | Is the consent form for sterilization read out and explained to the client in her language? | | | 🞏 Yes 🞏 No | | |
| **PP 1.4** | Client re-confirms decision to opt for sterilization? | | | 🞏 Yes 🞏 No | | |
| **PP 1.5** | Ensures consent form is signed or thumb impression given by the client? | | | 🞏 Yes 🞏 No | | |
| **Pause Point 2 (Pre-operative)** | | | | | | |
| **Provider IDs -** | | **Client ID -** | | | | |
| **PP 2.1** | **Appropriate infection prevention practices**  ***Observe and note down whether the service provider:*** | | |  | | |
| 1 | On entering the OT –  changed in to OT clothes, 🞏 Yes 🞏 No  OT slippers/shoes, 🞏 Yes 🞏 No  cap & 🞏 Yes 🞏 No  mask 🞏 Yes 🞏 No | | | 🞏 Yes 🞏 No | | |
| 2 | Surgical scrub (for surgeon & assistants) is performed as per norms and changes into sterile gown before beginning the procedure | | | 🞏 Yes 🞏 No | | |
| 3 | Uses a pair of fresh gloves for this case | | | 🞏 Yes 🞏 No | | |
| 4 | Uses sterilized (autoclaved)instruments for this case | | | 🞏 Yes 🞏 No | | |
| **PP 2.2** | **Client preparation for the surgery**  ***Observe and note down whether the service provider:*** | | |  | | |
| 1 | Ensures client has emptied her bladder just before beginning the procedure | | | 🞏 Yes 🞏 No | | |
| 2 | Provides sedation and analgesia using Inj. Fortwin and Phenargan . If not available, gives other appropriate drug/s. | | | 🞏 Yes 🞏 No  If yes, what was provided  🞏 Fortwin &  🞏 Phenargan  🞏 Other (Specify)  ________________ | | |
| 3 | 2% plain xylocaine was used after diluting with equal amounts of Normal Saline or Distilled Water (to make 1%). | | | 🞏 Yes 🞏 No | | |
| **Pause Point 3 (Intra-operative practices)** | | | | | | |
| **Provider IDs-** | | **Client ID -** | | | | |
| **PP 3.1** | **Incision site was scrubbed adequately**  ***Observe and note down whether the service provider adheres to following practices:*** | | |  | | |
| 1 | Antiseptic solution was applied twice to the incision area | | | 🞏 Yes 🞏 No | | |
| 2 | Abdomen was cleaned in a circular motion moving outwards from incision area | | | 🞏 Yes 🞏 No | | |
| 3 | In case of interval ligation, cleaned upper part of pubis and thighs as well. | | | 🞏 Yes 🞏 No  🞏 Not applicable | | |
| 4 | In case of postpartum ligation, cleaned the umbilicus first with an antiseptic soaked swab | | | 🞏 Yes 🞏 No  🞏 Not applicable | | |
| **PP 3.2** | **Sterile drapes were used for this client** | | | 🞏 Yes 🞏 No | | |
| **PP 3.3** | **Satisfactory anaesthetic effect checked for before proceeding further** | | | I🞏 Yes 🞏 No | | |
| **PP 3.4** | **Client's vitals monitored and documenetd during the surgery at least once**  ***(Observe and note down whether the service provider)*** | | |  | | |
|  | Monitored client’s BP | | | 🞏 Yes 🞏 No | | |
|  | Documented client’s BP | | | 🞏 Yes 🞏 No | | |
|  | Monitored client’s pulse | | | 🞏 Yes 🞏 No | | |
|  | Documented client’s pulse | | | 🞏 Yes 🞏 No | | |
| **PP 3.5** | **Recommended surgical technique for Minilap was followed**  ***(Observe and note down whether the service provider adheres to following practices)*** | | |  | | |
| 1 | Both fallopian tubes identified by tracing up to fimbrial end | | | 🞏 Yes 🞏 No | | |
| 2 | Isthmic portion of both fallopian tubes identified, transfixed and cut | | | 🞏 Yes 🞏 No | | |
| 3 | Catgut used for ligation | | | 🞏 Yes 🞏 No | | |
| **Pause Point 4 (Post-operative practices)** | | | | | | |
| **Provider IDs -** | | **Client ID -** | | | | |
| **PP 4.1** | **Client shifted from OT on a trolley or wheelchair** | | | 🞏 Yes 🞏 No | | |
| **PP 4.2** | **Minilap client monitored for at least four hours after surgery**  ***(Observe and note whether the service providers adhere to following practices)*** | | |  | | |
| 1 | BP and pulse rate are checked  🞏 BP 🞏 Pulse | | | 🞏 Yes 🞏 No | | |
| 2 | BP and pulse rate are documented  🞏 BP 🞏 Pulse | | | 🞏 Yes 🞏 No | | |
| 3 | Surgical dressing checked for soakage | | | 🞏 Yes 🞏 No | | |
| **PP 4.3** | **Routine follow-up protocol was explained to the client**  ***(Observe and note whether the service providers advise on)*** | | |  | | |
| 1 | 1st follow-up within 48 hours of discharge by Health worker/ Community worker | | | 🞏 Yes 🞏 No | | |
| 2 | 2nd follow-up on 7th day after surgery | | | 🞏 Yes 🞏 No | | |
| 3 | 3rd follow-up at facility after one month of surgery or next menstrual period (whichever is earlier) | | | 🞏 Yes 🞏 No | | |
| **PP 4.4** | **The duly filled client card/discharge slip has been given back to the client** | | | 🞏 Yes 🞏 No | | |
